# Consumptive Combatants

**Published:** 1918-01-26

**Authors:** 


### The Hospital

The Workers' Newspaper of Administrative Medicine and Institutional Life, Administration, National Insurance and Health.

No. 1651. Vol. LXIII. SATURDAY, JANUARY 26, 1918. PRICE ONE PENNY

### CONSUMPTIVE COMBATANTS.

The Local Government Board, in a letter to the County Council and County Borough Clerks, have promulgated the new arrangements for treatment of Service victims of pulmonary tuberculosis made necessary by the great increase in their number. The Insurance Commissioners, after consultation with the Local Government Board and the Ministry of Pensions, and taking powers under Section 4 of the Part I. Amendment of the National Insurance Act, have extended sanatorium benefit to all such invalided uninsured combatants (save officers and nurses, whose cases will be dealt with by the Ministry of Pensions) whose income does not exceed £160 per annum. Those who are passing rich on more than four times forty pounds a year will be seen to by the Ministry of Pensions also. The letter of the L.G.B. goes on to say that it is anticipated that insurance committees generally will be desirous of arranging with the councils of their areas for the provision of the necessary residential treatment for discharged phthisical sailors and soldiers; and that in aid of the immediate preferential treatment of such in residential institutions special grants are payable to insurance committees, with which to get from the local councils the necessary facilities. Also that where the tuberculosis officer or other medical adviser certifies residential treatment to be desirable, although not likely to be curative, such residential treatment will be paid for by the Ministry of Pensions. Lastly, that the Board would be glad to be informed whether the council have available any vacant accommodation which would be suitable for cases in this latter category.

It might have been said, in the former one too; for the provision of the great number of extra beds now needed is the crux of the whole matter, failing solution of which the new arrangements are but a formal expression of good will. The difficulties involved are very great. At a time like the present, with the building trade at a standstill, extra accommodation can only be of a more or less temporary nature. What is actually being done—for action has for some time been taken on the lines now formulated—is that, as in other countries, plenty of huts are used, into which the patients are moved from the main buildings as they become more convalescent. Let anyone try sleeping in an open-windowed hut one of these January nights, and he will find that some hardiness is needed. Soldiers, in particular, are apt to think they have shivered enough already in the field. However, these are not altogether new difficulties in sanatoriums, and patients led by a strong medical director—a Walther or a Paterson—have been known to sink their personal feelings and work out their own salvation in a most commendable way. But unfortunately, as we have often pointed out, the vast bulk of British public sanatoriums have been established on wrong principles. The resident medical man has had but the position of a glorified house surgeon, robbed of all prestige by visiting honoraries or a visiting tuberculosis officer. Two years was a long time for him to hold his post. So it comes that consumptive combatants are leaving public sanatoriums in droves. In a case recently reported to us, at a 200-bed institution, providing very largely for Service consumptives, over twenty patients were dismissed and over fifty left of their own accord in the space of three months. As ever, the time of trial reveals defects.

The hint to local councils to look for accommodation for the more advanced cases, to be paid for by the Ministry of Pensions, will probably be easier to respond to. These cases, being definitely invalids and probably often confined to bed, will be much more manageable. The accommodation required, too, will approximate much more nearly to that of the ordinary hospital or nursing home. It will, of course, be subject to final inspection, with regard to approval or rejection, by one of the Board's officials. Would that all tuberculosis arrangements and decisions of local bodies were so subject! The "centralised control" that Sir William Osler desiderates in this matter seems a pre-requisite to real efficiency. Decentralisation may be far easier; it may make a good appearance; Lord Rhondda, short of time and *personnel* for rationing, may "believe tremendously" in it; and, indeed, it may suit national habits of the last decade or two—but it will not get the better of the bacillus of Koch. Meantime, those who have deserved so well of their country must not be allowed to suffer from faults of administration. It is of no use to get through waiting-lists of consumptives by letting them make of the sanatorium a sort of week-end resort. Sanatorium superintendents must be consulted more directly as to this problem, must be given extended powers, privileges, and responsibilities. Theirs are the suggestions which are likely to be most valuable in affording some solution, a solution which is likely to vary in different localities. But new measures need to be initiated in addition to the drawing up of schemes on paper, if the ravages of war on the nation's manhood are to be coped with to anything like a desirable degree.

